# Anti‐SARS‐CoV‐2 spike immunoglobulin G and immunoglobulin M titers decline as interval from the second inactivated vaccine dose to the onset of illness is prolonged in breakthrough infection patients

**DOI:** 10.1111/crj.13590

**Published:** 2023-02-09

**Authors:** Chuan‐cai Xu, Zhi‐song He, Wei Lei, Jin‐zhou Zhu, Da‐guo Zhao, Jin‐dan Kong, Yao Wei, Ying Xu, Jian‐An Huang

**Affiliations:** ^1^ Department of Pulmonary and Critical Care Medicine the First Affiliated Hospital of Soochow University Suzhou China; ^2^ Department of Cardiology the First Affiliated Hospital of Soochow University Suzhou China; ^3^ Department of Gastroenterology the First Affiliated Hospital of Soochow University Suzhou China; ^4^ Department of Critical Care Medicine the First Affiliated Hospital of Soochow University Suzhou China; ^5^ Department of Infectious Diseases the First Affiliated Hospital of Soochow University Suzhou China

**Keywords:** anti‐SARS‐CoV‐2 spike IgG antibody, anti‐SARS‐CoV‐2 spike IgM antibody, IL‐6, inactivated vaccine, lymphocyte, SARS‐CoV‐2

## Abstract

**Background:**

Understanding of the early immune response in severe acute respiratory syndrome coronavirus 2 (SARS‐CoV‐2) breakthrough infections is limited.

**Methods:**

Ninety‐eight patients with coronavirus disease 2019 (COVID‐19) breakthrough infections were divided into two groups, with intervals from receiving the second dose of inactivated vaccine to the onset of illness <60 or ≥60 days.

**Results:**

The median lymphocyte count and the median anti‐SARS‐CoV‐2 spike immunoglobulin G (IgG) and immunoglobulin M (IgM) titers were higher in the <60‐day interval group compared with the corresponding medians in the ≥60‐day interval group (*p* = 0.005, *p* = 0.001, and *p* = 0.001, respectively). The median interleukin‐6 (IL‐6) level in the <60‐day interval group was significantly lower than the median IL‐6 level in the ≥60‐day interval group (*p* < 0.001).

**Conclusions:**

Our results highlight the different anti‐SARS‐CoV‐2 spike IgG and IgM antibody titers among patients with different intervals from receiving the second dose of inactivated vaccine to the onset of illness.

## Graphical abstract

The median titers of anti‐SARS‐CoV‐2 spike IgG and IgM and lymphocyte counts in the <60‐day interval group were significantly higher than that in the ≥60‐day interval group. The interval from receiving the second dose of inactivated vaccine to the onset of illness increases, the early immune response to breakthrough infection is weakened.

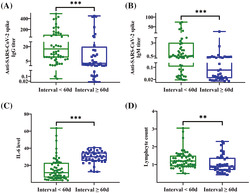

**Abbreviations**
COVID‐19: 2019 coronavirus disease
ct: cycle threshold
Hs‐CRP: hypersensitive C‐reactive protein
IgG: immunoglobulin G
IgM: immunoglobulin M
IL‐6: interleukin‐6
IQR: interquartile range
LDH: lactate dehydrogenase
RNA: ribonucleic acid
RT‐PCR: reverse transcription‐ polymerase chain reaction
SARS‐CoV‐2: severe acute respiratory syndrome coronavirus‐2
SD: standard deviation

## 1 INTRODUCTION

The severe acute respiratory syndrome coronavirus 2 (SARS‐CoV‐2) variant is prevalent around the world,^1^, ^2^ and the protection of vaccines against variants has waned.^3^, ^4^ The number of patients with coronavirus disease 2019 (COVID‐19) is surging, overwhelming hospital services. After vaccination, the gradual decrease of neutralizing antibodies overtime weakens host immunity against the virus.^5^ Therefore, the incidence of breakthrough infection remains high.

Real‐world studies in many countries have shown that vaccine effectiveness against SARS‐CoV‐2 infection is 86.1%–87% over 7 days after the second dose. The effectiveness drops to 77.5% 1 month after vaccination and 51.7% 4 months after vaccination.^4^, ^6^, ^7^ Due to immune escape of the variants,^8^, ^9^ breakthrough infection is unavoidable. The levels of SARS‐CoV‐2 specific antibodies after breakthrough infection reflect the ability of the host immune system to respond to the virus. However, little research on the levels of SARS‐CoV‐2 specific antibodies in the early stage after breakthrough infection has been reported.

For this study, clinical data from 98 breakthrough infection patients were collected, and the difference in anti‐SARS‐CoV‐2 spike antibody titers after infection in patients with different intervals from receiving the second dose of vaccine to the onset of illness was determined. This data will facilitate optimal clinical management of breakthrough infection patients.

## 2 METHODS

### 2.1 Data collection

The 98 SARS‐CoV‐2 breakthrough infection patients included in this study were hospitalized in the Third People's Hospital of Yangzhou from 28 July to 26 August 2021. All patients were fully vaccinated with inactivated vaccines (CoronaVac, Sinovac Life Sciences, Beijing, China, or BBIBP‐CorV, Beijing Institute of Biology, Beijing, China). SARS‐CoV‐2 diagnoses were confirmed with positive polymerase chain reaction tests (Liferiver Co. Ltd, Shanghai, China) from nasopharyngeal swabs. Clinical data of patients were extracted from the hospital database, including sex, age, body mass index, symptoms, and laboratory tests within 24 h of admission, such as SARS‐CoV‐2 nucleocapsid gene cycle threshold (ct) value, complete blood counts, serum biochemical parameters, interleukin‐6 (IL‐6), and specific serum immunoglobulin M (IgM) and immunoglobulin G (IgG) antibodies of SARS‐CoV‐2 receptor‐binding domain (Autobio Co. Ltd, Zhengzhou, China). A breakthrough infection was defined as a SARS‐CoV‐2 infection occurring at least 14 days from receiving the second dose of inactivated vaccine.^10^, ^11^ Patients were divided into two groups: with intervals from receiving the second dose of inactivated vaccine to the onset of illness of <60 or ≥60 days. Because this study was retrospective and non‐interventional, an ethics exemption was granted from the ethics committee of the Third People's Hospital of Yangzhou.

### 2.2 Anti‐SARS‐CoV‐2 spike IgG and IgM antibodies titer assays

Blood (3 mL) was collected in coagulation tubes from participants after fasting for 6 h. Blood samples were centrifuged at 3000 ×*g*, and the upper serum layer was analyzed for anti‐SARS‐CoV‐2 Spike IgG and IgM antibodies using a chemiluminescent particle immunoassay and the Autolumo A2000 plus system (Autobio Co. Ltd, Zhengzhou, China), according to the manufacturer's instructions.

### 2.3 Statistical analysis

Continuous variables are presented as mean ± standard deviation or median with interquartile range (IQR) and were analyzed by independent sample *t*‐tests or nonparametric Mann–Whitney *U* tests. Categorical variables were expressed as the number and percentage and compared using Chi‐square tests. Correlation analyses were performed using the Spearman method. Analyses were performed using IBM SPSS Statistics version 26.0 (IBM Corp., Armonk, NY, USA) or GraphPad Prism 8.0 (GraphPad, San Diego, CA, USA). *p* < 0.05 (two‐tailed) was considered statistically significant.

## 3 RESULTS

### 3.1 Patient characteristics

The demographic characteristics and laboratory data of the study participants are shown in Tables 1 and 2. Thirty‐eight (38.78%) patients were males, and 60 (61.22%) patients were females. The median age of the patients was 46 (IQR, 35–57.25) years. Patients presented most commonly with cough (36.73%) on admission, followed by fever (28.57%) and sore throat (15.6%). No symptoms (14.29%) and stuffy nose (8.2%) were also frequently observed. The median time from the onset of illness to hospital admission was 2 days (IQR, 1–3 days). Twenty‐one (21.43%) patients had comorbidities. Common comorbidities included hypertension (17 cases, 17.35%), diabetes (six cases, 6.12%), and coronary heart disease (four cases, 4.08%). The median duration of viral RNA shedding was 12.27 ± 4.59 days. The average ct value was 30.48 ± 6.19.

**TABLE 1:** Demographic characteristics of all 98 patients.

	Total (*n* = 98)	Interval <60 d (*n* = 58)	Interval ≥60 d (*n* = 40)	*p* *
Gender				0.53
Male, No. (%)	38 (38.78)	21 (36.21)	17 (42.5)	
Female, No. (%)	60 (61.22)	37 (63.79)	23 (57.5)	
Age, years	46 (35–57.25)	47 (35–58)	43.5 (37–55)	0.772
BMI, kg/m^2^	23.55 ± 2.14	23.71 ± 2.4	23.32 ± 1.67	0.342
Smoking, No. (%)	37 (37.76)	20 (34.48)	17 (42.5)	0.421
Comorbidities, No. (%)	21 (21.43)	15 (25.86)	6 (15)	0.198
Hypertension, No. (%)	17 (17.35)	13 (22.41)	4 (10)	
Diabetes, No. (%)	6 (6.12)	4 (6.9)	2 (5)	
Coronary heart disease, No. (%)	4 (4.08)	4 (6.9)		
Chronic pulmonary disease, No. (%)	1 (1.02)		1 (2.5)	
Chronic liver disease, No. (%)	1 (1.02)	1 (1.72)		
Hyperthyreosis, No. (%)	1 (1.02)		1 (2.5)	
History of cerebral hemorrhage, No. (%)	1 (1.02)	1 (1.72)		
Days from illness onset to hospital admission	2 (1–3)	2 (1–3)	2 (1–3)	0.286
Viral shedding duration after illness onset (days)	12.27 ± 4.59	12.07 ± 4.42	12.55 ± 4.87	0.698

*Note*: Continuous variables were presented as mean ± standard deviation or median with interquartile range and analyzed by independent sample *t*‐tests or nonparametric Mann–Whitney *U* tests. Categorical variables were expressed as the number and percentage and compared using Chi‐square tests.

Abbreviation: BMI, body mass index.

* *p* value: comparisons of the <60‐day interval and the ≥60‐day interval groups.

**TABLE 2:** Laboratory tests of all 98 patients.

	Total (*n* = 98)	Interval <60 d (*n* = 58)	Interval ≥60 d (*n* = 40)	*p* *
ct value	30.48 ± 6.19	30.97 ± 5.4	29.77 ± 7.21	0.375
Anti‐SARS‐CoV‐2 spike IgG titer	11.22 (2.29–46.23)	17.62 (8.88–102.73)	2.75 (0.5–20.04)	**0.001**
Anti‐SARS‐CoV‐2 spike IgM titer	0.29 (0.04–1.7)	0.52 (0.11–3.04)	0.08 (0.03–0.38)	**0.001**
IL‐6, pg/mL	22.69 (9–30.75)	11.4 (5.75–22.9)	29.96 (25.3–34.91)	<**0.001**
LYM, ×10^9^/L	1.02 (0.89–1.42)	1.2 (0.9–1.5)	0.91 (0.7–1.38)	**0.005**
D‐dimer, mg/L	0.37 (0.2–0.52)	0.3 (0.2–0.51)	0.4 (0.27–0.55)	0.313
LDH, IU/L	189 (161–230.5)	190.5 (162–229.25)	186.5 (158.25–234.25)	0.761
Hs‐CRP, mg/L	9.24 (1.55–30.38)	10.7 (3.78–28.38)	7.42 (0.7–34.89)	0.356

*Note*: Laboratory tests were obtained within 24 h of admission. Continuous variables were presented as mean ± standard deviation or median with interquartile range and analyzed by independent sample *t*‐tests or nonparametric Mann–Whitney *U* tests. Values in bold indicate that the difference in the data in this row is statistically significant.

Abbreviations: ct, cycle threshold; Hs‐CRP, high sensitivity c‐reactive protein; IgG, immunoglobulin G; IgM, immunoglobulin M; IL‐6, interleukin‐6; LDH, lactate dehydrogenase; LYM, lymphocyte count; SARS‐CoV‐2, severe acute respiratory syndrome coronavirus‐2.

* *p* value: comparisons of the <60‐day interval and the ≥60‐day interval groups.

### 3.2 Laboratory tests

**FIGURE 1:**
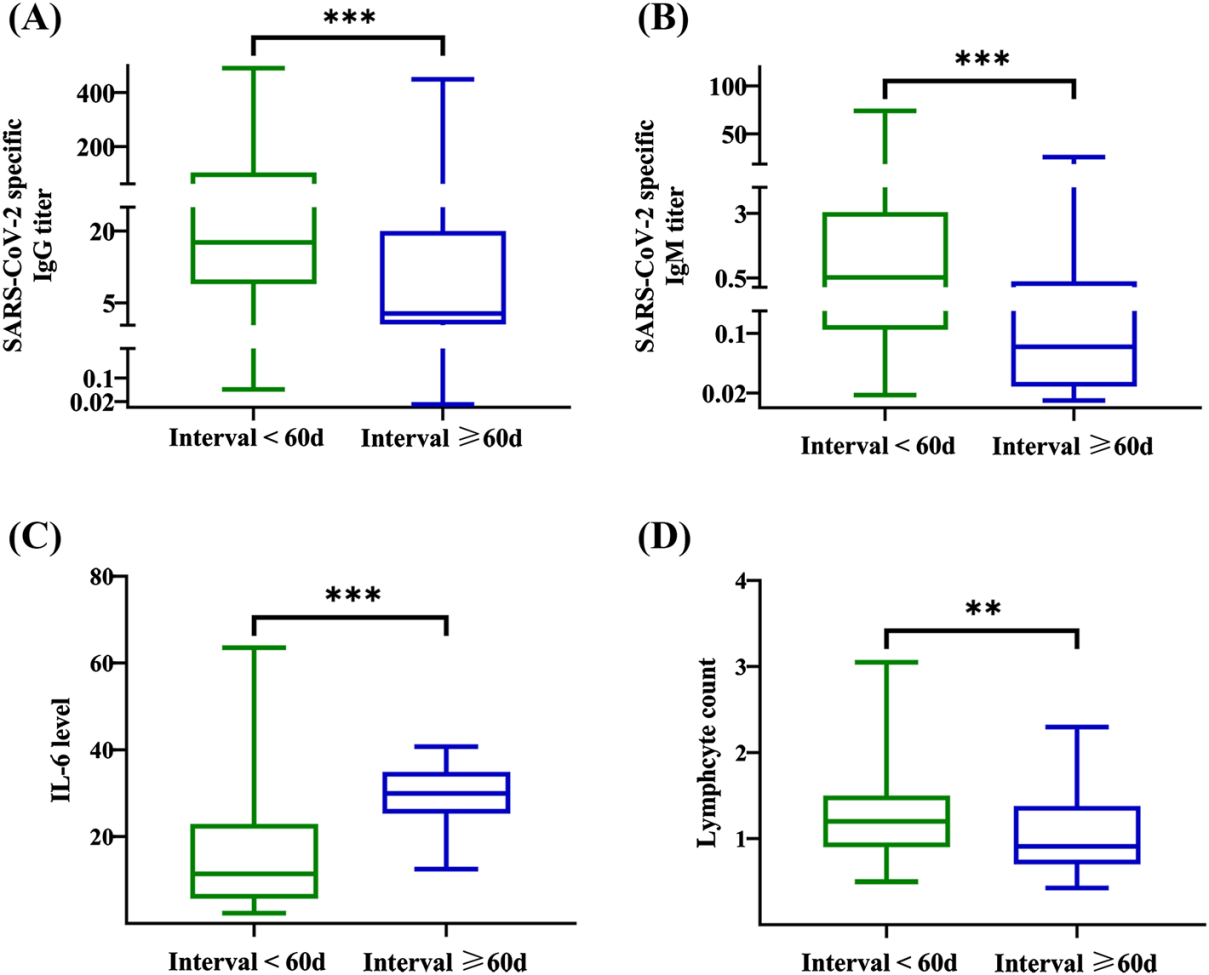
Anti‐SARS‐CoV‐2 spike IgG and IgM titers, IL‐6 levels, and lymphocyte counts in patients with intervals from receiving the second dose of inactivated vaccine to the onset of illness of <60 or ≥60 days. (A) The median titer of anti‐SARS‐CoV‐2 spike IgG was significantly higher in the <60‐day interval group compared with the IgG titer in the ≥60‐day interval group. (B) The median titer of anti‐SARS‐CoV‐2 spike IgM was significantly higher in the <60‐day interval group compared with the IgM titer in the ≥60‐day interval group. (C) The median IL‐6 level in the <60‐day interval group was significantly lower compared with the median IL‐6 level in the ≥60‐day interval group. (D) The median lymphocyte count in the <60‐day interval group was significantly higher compared with the median lymphocyte count in the ≥60‐day interval group. Continuous variables of non‐normal distribution were expressed as median with interquartile range (IQR) and analyzed with nonparametric Mann–Whitney *U* tests. Abbreviations: IgG, immunoglobulin G; IgM, immunoglobulin M; IL‐6, interleukin‐6; IQR, interquartile range; SARS‐CoV‐2, severe acute respiratory syndrome coronavirus‐2. **p < 0.01. ***p < 0.001.

The median anti‐SARS‐CoV‐2 spike IgG and IgM titers were higher in the <60‐day interval group (17.62 S/co, IQR, 8.88–102.73 and 0.52 S/co, IQR, 0.11–3.04, respectively) compared with the titers in the ≥60‐day interval group (2.75 S/co, IQR, 0.5–20.04 and 0.08 S/co, IQR, 0.03–0.38, respectively) (*p* = 0.001). The median IL‐6 level in the <60‐day interval group (11.4 pg/mL, IQR, 5.75–22.9) was significantly lower than the median IL‐6 level in the ≥60‐day interval group (29.96 pg/mL, IQR, 25.3–34.91) (*p* < 0.001). The median lymphocyte count was significantly higher in the <60‐day interval group (1.2 × 10^9^/L, IQR, 0.9–1.5) compared with the median lymphocyte count in the ≥60‐day interval group (0.91 × 10^9^/L, 0.7–1.38) (*p* = 0.005) (Figure 1).

Spearman correlation analysis showed that anti‐SARS‐CoV‐2 spike IgG and IgM titers inversely correlated with IL‐6 levels (*r* = −0.359, *p* < 0.001 and *r* = −0.325, *p* = 0.001, respectively) and positive correlated with lymphocyte counts (*r* = 0.283, *p* = 0.005 and *r* = 0.251, *p* = 0.013, respectively). Lymphocyte counts inversely correlated with IL‐6 levels (*r* = −0.287, *p* = 0.004) (Supplementary Tables S1, S2, and S3).

## 4 DISCUSSION

Our results demonstrate that anti‐SARS‐CoV‐2 spike IgG and IgM titers in patients receiving the second dose of inactivated vaccine ≥60 days from the onset of illness were significantly lower than those in the patients receiving the second dose of inactivated vaccine <60 days from the onset of illness.

In a recent study, 6 months after vaccination, neutralizing antibody levels decreased to varying degrees.^5^ Breakthrough infections were often associated with low neutralizing antibody titers.^12^ Compared with the first 90 days after the second vaccine dose, the risk of infection in all age groups was 2.37 times higher 90–119 days after vaccination, 2.66 times higher 120–149 days after vaccination, 2.82 times higher 150–179 days after vaccination, and 2.82 times higher 180 days or more after vaccination.^13^ Because breakthrough infections caused by variants are unavoidable, the early immune response of the host after infection is crucial. In our study, anti‐SARS‐CoV‐2 spike IgG and IgM antibody titers were determined after infection, and the median time from onset to the admission of all patients was 2 days. Therefore, the IgG and IgM antibody titers may represent the patient's ability to develop an early serological response to breakthrough infection. This suggests that as the interval from receiving the second dose of inactivated vaccine to the onset of illness increases, the early immune response to breakthrough infection is weakened.

Total receptor‐binding domain‐specific immunoglobulin levels were significantly increased in vaccinated patients after breakthrough infection.^14^ In addition, the mRNA vaccine can boost the host immune system's response to infection.^15^ This may explain how vaccination reduces the risk of hospitalization, severe illness, and death.^4^, ^6^, ^7^

We demonstrated that lymphocyte counts were low and IL‐6 levels were high in patients whose intervals from receiving the second dose of inactivated vaccine to the onset of illness were ≥60 days. Low lymphocyte counts are associated with disease severity and mortality.^16^, ^17^ The presence and severity of lymphopenia is a reliable predictive marker of poor prognosis.^18^ A multivariate logistic analysis showed that lymphocyte counts and IL‐6 levels were independent factors predicting severe SARS‐CoV‐2.^19^

A randomized clinical trial showed that recombinant human granulocyte colony‐stimulating factor treatment reduced the number of COVID‐19 patients with lymphopenia developing critical illness or death.^20^ IL‐6 is a useful marker for discriminating severe illness from mild to moderate COVID‐19.^17^ Many studies showed that patients with severe COVID‐19 have higher IL‐6 levels than those with moderate illness.^16^, ^21^ In addition, high serum IL‐6 levels are significantly associated with adverse clinical outcomes, including acute respiratory distress syndrome (ARDS), invasive mechanical ventilation, ICU admission, and death.^17^, ^22^ IL‐6 is a critical mediator of shock and multiple organ dysfunction syndrome in critically ill patients with COVID‐19.^23^ According to Mazzoni et al.,^16^ high IL‐6 levels lead to impaired immune cytotoxic activity in lymphocytes, which are necessary to fight viral illnesses.

In our study, lymphocyte counts negatively correlated with serum IL‐6 levels. Increased lymphocyte counts were observed after tocilizumab (an anti‐IL‐6 receptor antibody) treatment in several studies.^16^, ^23^, ^24^ Sarilumab, an IL‐6 blocker, also significantly affected severe COVID‐19.^25^ We also showed that SARS‐CoV‐2 specific IgG and IgM antibody titers negatively correlated with IL‐6 levels and positively correlated with lymphocyte counts. A dysregulated host immune response to SARS‐CoV‐2 and cytokine storm syndrome contributes to the development of organ dysfunction and is a major factor in morbidity and mortality.^26^ Therefore, blocking IL‐6 is very important in reducing inflammation, especially in severe and critically ill patients.

This study had several limitations. First, this study involved a small number of cases and was retrospective. Second, because few severely or critically ill patients in Yangzhou have been vaccinated, only one severely ill patient was included in our study.

## 5 CONCLUSION

In conclusion, our study provides new insights into the early immune response to breakthrough infection in patients with COVID‐19, highlighting different IgG and IgM antibody titers among patients with different intervals from receiving the second dose of inactivated vaccine to the onset of illness.

## AUTHOR CONTRIBUTIONS

Chuan‐cai Xu, Zhi‐song He, and Wei Lei contributed to the study design and had full access to all data in the study. Chuan‐cai Xu and Zhi‐song He collected patient samples; Chuan‐cai Xu and Wei Lei analyzed the data; Chuan‐cai Xu and Zhi‐song He drafted the manuscript; Ying Xu and Jian‐an Huang reviewed the manuscript. Jin‐zhou Zhu, Jin‐dan Kong, Yao Wei, and Da‐guo Zhao contributed to data acquisition, data analysis, or data interpretation. All authors read and approved the final manuscript.

## CONFLICT OF INTEREST

The authors declare no competing interests in this study.

## ETHICS APPROVAL AND CONSENT TO PARTICIPATE

Our research was granted an ethics exemption from the ethics committee of the Yangzhou Third People's Hospital for it was retrospective and non‐interventional. The study was based on existing data collected in the course of routine clinical practice, and no additional risks are posed to patients. Therefore, the individual's informed consent was waived by the above ethics committee. Due to the highly transmissible feature of COVID‐19, in order to avoid the potential cross‐infection, verbal informed consent was obtained from all patients, and the text of each patient's verbal consent was recorded by the investigator in the patient's medical records. The study was performed in accordance with the Declaration of Helsinki.

## Supporting information


**Table S1.** SARS‐CoV‐2 specific IgG and IgM titers inversely correlated with IL‐6 level.
**Table S2.** SARS‐CoV‐2 specific IgG and IgM titers correlated with lymphocyte count.
**Table S3.** IL‐6 level inversely correlated with Lymphocyte count.

## Data Availability

Additional data can be requested from the corresponding author.
