# Supplementary material for: Anti‐SARS‐CoV‐2 spike immunoglobulin G and immunoglobulin M titers decline as interval from the second inactivated vaccine dose to the onset of illness is prolonged in breakthrough infection patients
Source: Clin Respir J. 2023 Feb 9;17(4):270–6. doi: 10.1111/crj.13590 (PMC10113280; doi:10.1111/crj.13590)
Supplement: Supplementary file 1 — Table S1. SARS‐CoV‐2 specific IgG and IgM titers inversely correlated with IL‐6 level. Table S2. SARS‐CoV‐2 specific IgG and IgM titers correlated with lymphocyte count. Table S3. IL‐6 level inversely correlated with Lymphocyte count. [file CRJ-17-270-s001.docx]

**Supplementary Table 1. SARS-CoV-2 specific IgG and IgM titers inversely correlated with IL-6 level**

|  | **IL-6** | ***P*** |
| --- | --- | --- |
| **SARS-CoV-2 specific IgG titer** | r = −0.359 | 0 |
| **SARS-CoV-2 specific IgM titer** | r = −0.325 | 0.001 |

Correlation analyses were performed using the Spearman method. Spearman correlation coefficient was represented by the r value. *P* value of less than 0.05 (two-tailed) was considered statistically significant.

Abbreviations: SARS-CoV-2, severe acute respiratory syndrome coronavirus-2; IgG: immunoglobulin G; IgM: immunoglobulin M; IL-6, interleukin-6.

**Supplementary Table 2. SARS-CoV-2 specific IgG and IgM titers correlated with lymphocyte count**

|  | **lymphocyte count** | ***P*** |
| --- | --- | --- |
| **SARS-CoV-2 specific IgG titer** | r = 0.283 | 0.005 |
| **SARS-CoV-2 specific IgM titer** | r = 0.251 | 0.013 |

Correlation analyses were performed using the Spearman method. Spearman correlation coefficient was represented by the r value. *P* value of less than 0.05 (two-tailed) was considered statistically significant.

Abbreviations: SARS-CoV-2, severe acute respiratory syndrome coronavirus-2; IgG: immunoglobulin G; IgM: immunoglobulin M.

**Supplementary Table 3. IL-6 level inversely correlated with Lymphocyte count**

|  | **IL-6** | ***P*** |
| --- | --- | --- |
| **Lymphocyte count** | r = −0.287 | 0.004 |

Correlation analyses were performed using the Spearman method. Spearman correlation coefficient was represented by the r value. *P* value of less than 0.05 (two-tailed) was considered statistically significant.Abbreviation: IL-6, interleukin-6
